# Health Behaviors and Overweight in Nursing Home Employees: Contribution of Workplace Stressors and Implications for Worksite Health Promotion

**DOI:** 10.1155/2015/915359

**Published:** 2015-08-25

**Authors:** Helena Miranda, Rebecca J. Gore, Jon Boyer, Suzanne Nobrega, Laura Punnett

**Affiliations:** ^1^Department of Work Environment & Center for the Promotion of Health in the New England Workplace (CPH-NEW), University of Massachusetts Lowell, Lowell, MA 01854, USA; ^2^School of Health Sciences, University of Tampere, 33014 Tampere, Finland; ^3^Boston Children's Hospital, Boston, MA 02115, USA

## Abstract

*Background*. Many worksite health promotion programs ignore the potential influence of working conditions on unhealthy behaviors. *Methods*. A study of nursing home employees (56% nursing aides) utilized a standardized questionnaire. We analyzed the cross-sectional associations between workplace stressors and obesity, cigarette smoking, and physical inactivity. *Results*. Of 1506 respondents, 20% reported exposure to three or more workplace stressors (physical or organizational), such as lifting heavy loads, low decision latitude, low coworker support, regular night work, and physical assault. For each outcome, the prevalence ratio was between 1.5 and 2 for respondents with four or five job stressors. Individuals under age 40 had stronger associations between workplace stressors and smoking and obesity. *Conclusions*. Workplace stressors were strongly associated with smoking, obesity, and physical inactivity, even among the lowest-status workers. Current working conditions affected younger workers more than older workers. Although this study is cross-sectional, it has other strengths, including the broad range of work stressors studied. Strenuous physical work and psychosocial strain are common among low-wage workers such as nursing home aides. Workplace health promotion programs may be more effective if they include measures to reduce stressful work environment features, so that working conditions support rather than interfere with employee health.

## 1. Introduction

Obesity, smoking, and physical inactivity represent important and preventable health risks. They are typically framed as the result of individual “lifestyle” choices and often targeted through health promotion programs that seek to motivate individual behavior change. A common venue for these programs is the workplace. However, workplace health promotion (WHP) programs sometimes suffer from low participation and uneven results, especially for low-income workers [1]. There are various possible reasons, including time and financial constraints and failure to incorporate a systems approach into program design [2–6].

In addition, working conditions themselves may contribute to individuals' unhealthy behaviors. For example, obesity has been linked with night work, long work hours, psychosocial job strain, and job insecurity [7–17]. Physical inactivity during leisure hours has been associated with low decision latitude (both in passive jobs and those with high strain) and frequent involuntary overtime [13, 18–24]. In some populations, smoking habits have been positively correlated with high job demands and job strain and negatively with resources at work (including job control), social support from coworkers and supervisors, and low social capital at work, a construct that overlaps with both social support and participation in decision-making [13, 23, 25–30].

Nonetheless, the literature is inconsistent on all of these associations. Among possible reasons both the lack of formal theoretical hypotheses and, somewhat in contrast, an incomplete set of risk factors are suggested (possibly stemming from excessive reliance on* a priori* models in this relatively early stage of accruing evidence) [9, 16]. Many of these studies have analyzed data from the general population, where the separate contributions of working conditions and socioeconomic status (SES) may be difficult to disentangle, even with multivariable statistical methods. Further, potential effect modification within these associations has been largely ignored, with the exception of comparing risks between men and women.

Limited decision-making at work is a dominant feature of jobs that are low in the organizational hierarchy, and lower SES workers are typically more exposed to other physical and psychosocial workplace stressors [31, 32], as well as risks from other aspects of their physical and social environment [33]. As a result they may experience additive or even synergistic health effects. However, the cooccurrence of these hazards has rarely been taken into account.

It is well-established that inactivity, obesity, and a variety of chronic diseases become more common with age in the general population. Thus it would be of value to understand better the trajectory of risk and its determinants over the life course. Yet potential effect modification by age on the association between work and health behaviors has been studied with surprising rarity.

This study was a part of a larger project (“ProCare”) examining a variety of factors influencing employee health in a large chain of skilled nursing facilities providing long-term care [34–38]. The purpose of the present analyses was to investigate the exposures of nursing home workers to physical and organizational stressors, whether these work exposures were associated with personal health risks, and (if so) whether the associations differed by age.

## 2. Methods

### 2.1. Study Population and Procedures

Questionnaires were distributed to employees in 18 nursing homes, in several states of the U.S., within a single company in 2006–2009. The surveys were timed relative to the implementation of a Safe Resident Handling program. In 12 centers, baseline surveys were administered during the week of initial training for department heads (defined as the implementation date), just prior to installation of the resident handling equipment. In the other 6 centers, the first surveys were conducted at least one year after program implementation, using a very similar instrument. The first (or “entry”) survey in each center was selected for these cross-sectional analyses.

All permanent full- and part-time clinical employees were eligible to participate. Clinical employees included nursing aides (NA), licensed practical nurses (LPN), and registered nurses (RN) as well as other direct care personnel such as physical and occupational therapists. In addition, office, laundry, food service, and janitorial staff were recruited for follow-up surveys in four centers, where a participatory WHP program was under consideration for the entire workforce.

Questionnaires were distributed at the nursing homes by members of the study team and completed by most workers during scheduled break times. For those who could not be met in person, such as third-shift and weekend employees, a prestamped, addressed return envelope was provided. Employees who returned the completed survey with the informed consent form received compensation of $20. The study proposal was approved by the Institutional Review Board of the University of Massachusetts Lowell (protocol #06-1403).

The self-administered questionnaire collected detailed information on demographic characteristics (e.g., age, gender, length of education, and ethnic origin), working conditions, health behaviors, and health status. To the extent possible, questions were derived from preexisting, validated items and scales.

### 2.2. Health Behaviors and Obesity

There were three outcome variables. Physical exercise was assessed by a single question: “how many times a week on average do you work up a sweat (at least 20 min per session, e.g., fast walking, jogging, bicycling, swimming, rowing, etc.)?” Response categories were none; some but less than once a week; 1–3 times per week; more than 3 times per week. Physical inactivity (yes/no) was defined as “none” versus any. Smoking was categorized as current, former, or never. Body mass index (BMI) was computed from self-reported weight and height; “obese” was defined as BMI of 30.0 or above.

### 2.3. Work Environment Characteristics

The questionnaire addressed psychological demands of work, job control, coworker support, and supervisor support (2 items each, from the Job Content Questionnaire (JCQ)) [39]; adequate staffing (1 item: “my work area is adequately staffed”); schedule control [40] (2 items); and regular night shift work (1 question). Workplace safety and climate issues included perceived safety (4 items, 2 from Griffin and Neal [41] and 2 developed by the investigators); having been assaulted at work by a resident, resident's visitor or family member in the past 3 months; and tolerance of discrimination (1 item: “this organization practices zero tolerance for discrimination”). Respondents were also asked about work-family interference [42] (3 items), employer support for family or other personal responsibilities (1 item), and other paid jobs outside the survey workplace. Physical requirements at work were characterized in terms of moving or lifting heavy loads (1 item, JCQ); rapid and continuous physical activity (1 item, JCQ); and awkward postures (3 items, JCQ). The sum of these 5 exposures was labeled “physically demanding work.”

All survey items were assessed with a 4-point Likert scale (strongly disagree; disagree; agree; strongly agree) and were dichotomized for these analyses between “disagree” and “agree.” For multi-item scales, the sum of the items (after reversing where appropriate) was dichotomized so as to create categories corresponding as closely as possible to the average of the original item distributions (e.g., if 22% of the workers replied “agree” or “strongly agree” to the first item, and 18% to the second item, their sum was dichotomized so that 20% agreed).

### 2.4. Statistical Analysis

The three outcome variables were the health behaviors of smoking and physical inactivity, plus obesity. The prevalences of the outcomes and the workplace stressors were compared by job title, geographical region (southern versus northern East Coast), and age group (under 40 years versus 40 or older).

Associations between outcomes and workplace stressors were assessed by cross-tabulation and log-binomial regression to estimate prevalence ratios (PRs) with 95% confidence intervals (CIs). If the log-binomial model failed to converge, the COPY method was used [43]. To limit the number of independent variables in the models, the five stressors with the highest crude associations with each outcome were chosen to construct an index with 5 levels (exposed to 0, 1, 2, 3, or 4-5 of the factors) for subsequent modeling. All models included gender, geographical region, education, and age (unless age-stratified). Tests of linear trend in effect with the exposure index were obtained by weighted linear regression of the model coefficients on the number of stressors, weighting by the inverse of the standard error of the coefficient.

There was some variation in outcomes by race/ethnicity but no confounding of the associations with exposure indices, so the models did not include ethnicity. The proportion of missing values in each variable in the analyses was at most 4%. All statistical analyses used the statistical software package SAS (version 9.1, SAS Institute Inc, Cary, NC, USA).

## 3. Results

### 3.1. Response, Demographics, and Job Characteristics

Questionnaires were received from 1,506 persons, of whom about 56% were nursing aides (Table 1). Response rate for the clinical staff was about 72% of the complete workforce rosters.

The age, gender, and race distributions were all consistent with the workforce demographics for these workplaces. Survey respondents were 89% female. Almost half (47%) were white, non-Latino, with a large difference by region: more African-American or African (67%) in the states farther south and a majority white in more northern states. The average age was 41 years (standard deviation (SD) 13); nursing aides were about 5 years younger than other employees, on average (Table 1). The mean length of work in the same type of job was 11 years (SD 10), although one in four workers reported over 17 years seniority. Lifetime experience in similar work (from questionnaires) was 6 to 8 years more than seniority in the current job (from workforce rosters). In the 4 centers where nonclinical workers were recruited they were slightly underrepresented (34% of all employees, 20% of respondents).

Survey respondents were 34% obese, 24% currently smoking, and 23% physically inactive outside of work. One in five held at least one other paid job. Employees reported high psychological demands at work (88% of respondents), awkward postures (65%), poor safety climate (60%), lifting heavy loads (57%), and work-family imbalance (43%). The prevalence of these stressors did not differ importantly by job title, although aides reported more physically heavy and psychologically demanding work, more recent assaults at work, and lower employer support for family responsibilities compared to all other workers combined (Table 1). There were moderate to high correlations among many of these factors [38].

Workers younger than 40 consistently reported more workplace stressors than those aged over 40 (Table 2), especially physical workload and safety problems. No major differences were seen in smoking or inactivity between the age groups, but older individuals were more likely to be obese.

### 3.2. Work Environment, Health Behaviors, and Obesity

Associations between the study outcomes and the separate work stressors were generally modest when examined separately, although there were many trends in the expected directions, that is, worse health behaviors with more stressful working conditions. In age-stratified cross-tabulations, associations were somewhat stronger among younger participants, the largest differences being for physical demands with smoking and night work with obesity. In contrast, the association between violence and physical inactivity was stronger among older than younger individuals.

**Figure 1:**
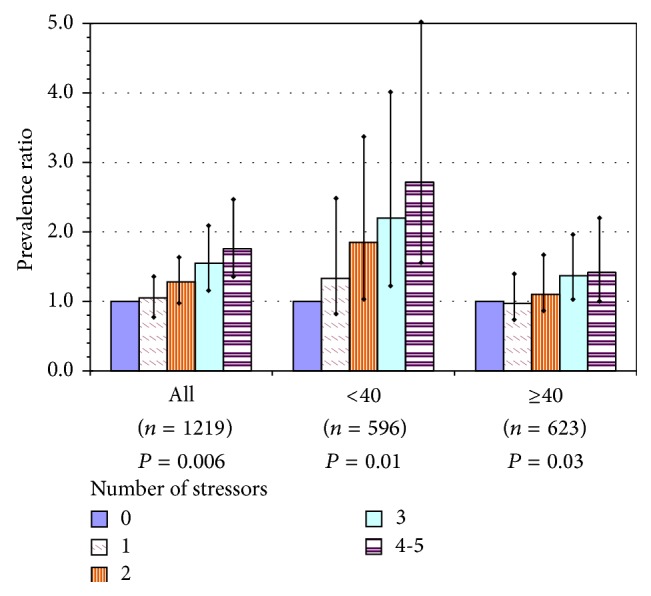
Overweight or obesity among U.S. nursing home employees, as a function of number of workplace stressors in the current job, for all participants and by age group: prevalence ratios with 95% confidence intervals for each level above 0 stressors and *P* value for test of linear trend. Index = sum of workplace stressors: poor coworker support, low decision latitude, recent assault(s) at work, work at night, and lifting heavy loads. Models adjusted for gender, education, and region; adjusted for age only in model of all workers.

The risk of obesity was linearly associated in multivariable modeling with the sum of these occupational features: low decision latitude, low coworker support, lifting heavy loads, night work, and recent physical assault. Twelve percent were not exposed to any of these stressors, whereas 27% were exposed to three or more and 8% to all five. The prevalence ratio was 1.8 for workers exposed to four or five stressors, compared to none; among nursing aides alone the PR was 2.0. Age strongly modified the risks, which were higher for younger workers (Figure 1).

**Figure 2:**
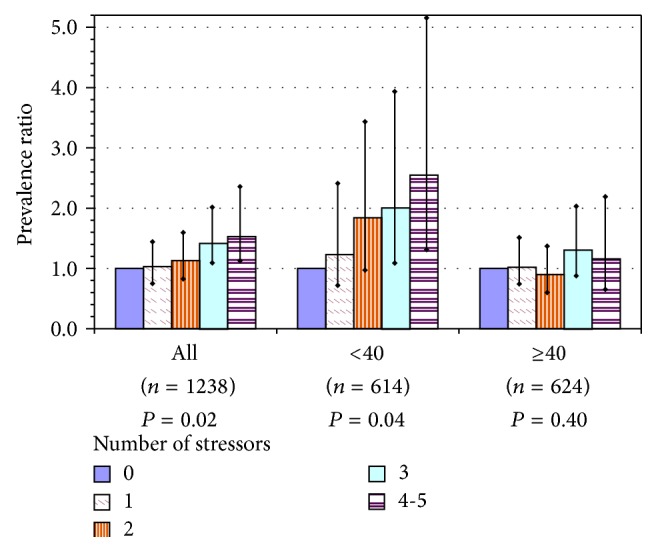
Current smoking among U.S. nursing home employees, as a function of number of workplace stressors in the current job, for all participants and by age group: prevalence ratios with 95% confidence intervals for each level above 0 stressors and *P* value for test of linear trend. Index = sum of workplace stressors: low decision latitude, low supervisor support, recent assault(s) at work, having another paid job, and physically demanding work. Models adjusted for gender, education, and region; adjusted for age only in model of all workers.

Current smoking was almost twice as high among workers exposed to at least 3 of 5 job stressors: low decision latitude, low supervisor support, having another paid job, physically demanding work, and recent physical assault (18% of workers). The effect was about the same when estimated for aides alone and much stronger among younger workers, with PRs of 2 and 2.5 for those with 3 and 4-5 exposures, respectively (Figure 2).

**Figure 3:**
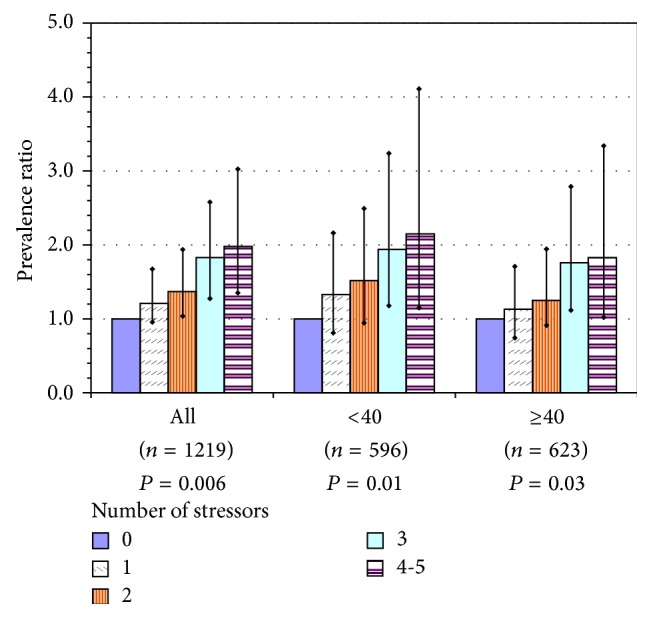
Physical inactivity among U.S. nursing home employees, as a function of number of workplace stressors in the current job, for all participants and by age group: prevalence ratios with 95% confidence intervals for each level above 0 stressors and *P* value for test of linear trend. Index = sum of workplace stressors: poor coworker support, low decision latitude, employer toleration of discrimination, work-family imbalance, and work at night. Models adjusted for gender, education, and region; adjusted for age only in model of all workers.

Physical inactivity showed the strongest trend with work stressors, of the three outcomes. The associated exposures were low decision latitude, low coworker support, employer tolerance of discrimination in the workplace, work-family imbalance, and night work. Of all workers, 21% reported 3 or more stressors. The risk of being inactive was approximately 2-fold for workers with 3 or more stressors, compared to none; the linear trend was similar for aides alone and varied little between the two age groups (Figure 3).

## 4. Discussion

### 4.1. Study Findings and Interpretations

Our survey results were consistent with a broader literature that the long-term care sector is a physically and psychologically demanding work environment [44–50], with a high frequency of physical assault on clinical staff [51–53]. Certified nursing assistants and other aides, who make up more than one-half of this workforce, experience particularly high physical and psychosocial workload.

Less appreciated to date is the extent to which these working conditions affect workers' “lifestyle.” In this study, the cooccurring workplace stressors had a linear association with health behaviors so that the higher the number of challenging working conditions, the more likely the reports of unhealthy behaviors. The risks were about two times higher for those exposed to 3 or more stressors, which applied to one-fifth of all workers. While shiftwork and low decision latitude have previously been linked to health behaviors, some of the contributing exposures in this population, physical workload, assault, work-family imbalance, and perceived toleration of discriminatory behavior, have not previously been reported.

The present findings are consistent with other evidence for the effect on health behaviors of psychosocial strain, night work, and low social support at work, although as noted above the literature is inconsistent to date and in fact discussions of some of these have been quite vigorous (e.g., [54–57]). There are many possible reasons for the inconsistent findings, related both to methodologic approaches and the joint distributions of independent variables within study populations. These issues cannot be resolved within the scope of the present work, only reflected upon in the effort to judge how to interpret these findings.

Among plausible reasons for disagreement, there may be a previous lack of attention to effect modification of some exposures by others, for example, if assault or night work is more potent in low-control jobs. It is common to “adjust for” numerous risk factors in multivariable regression modeling. However, to the extent that these are associated with each other, overadjustment may dilute the risk ratios for causally important variables. The choice of which domain is primary (for example, does SES explain away the effect of job exposures or vice versa?) reflects* a priori* beliefs about mechanism of effect that should be acknowledged explicitly not buried within modeling decisions. Examining associations within SES strata is a simple way to determine both the range of exposures within those strata and whether or not the effect is seen in each subgroup. At the very least these associations deserve continuing investigation to understand better the range of mechanisms that might operate in different settings and subgroups.

Further, the specific operationalization of conceptual domains may have great influence on the findings. For example, in one study, high job control was associated with leisure-time physical activity, whereas job demands alone had no such association [19].

Another issue in this multifactorial web is the possibility that different individuals express the strain through different health behaviors: some may self-medicate with food, while others smoke or drink more as a way to cope with stress and fatigue. Of note, a few investigators have taken the useful approach of examining the effect of job strain on multiple health risks simultaneously, to understand more fully the nature of the effect across parallel outcomes (e.g., [54, 58]).

Our study revealed age differences that have not been reported before: obesity and smoking had considerably stronger associations with workplace hazards among younger workers than among older ones. In fact, in the few prior analyses of effect modification by age in this area, other investigators have reported opposite trends from the present findings. Among Finnish nurses, those who consistently worked nights or rotating shifts both smoked more and were more often overweight than day workers. However, both of these effects interacted positively with age, so that nurses over 45 years had larger attributable increases [12]. Similarly, among men working on offshore oil/gas installations, higher age predicted higher BMI [59].

We cannot explain the discrepancy, but our findings show an effect in the opposite direction for both obesity and smoking. These results may be affected by “healthy worker” selection: the median turnover rate among all direct care staff in in U.S. nursing homes is 50% [60] and the high rate may be related to stressful working conditions [38]. Hence, those older workers who remained in their jobs may be more adapted to their working conditions or may have developed other coping strategies for work-related stress than smoking or comfort eating. Alternatively, increased work experience and age might be protective against these risks among older workers, but it seems more likely that weight, exercise, and smoking behaviors become more difficult to change later in life. If our results are valid, they have implications for the public health importance of improving the work environment in nursing homes, as the impact on the health behaviors of younger workers would have long-term benefits over the full course of their lives, including after retirement.

There are likely multiple mechanisms by which stressful features of the work environment influence health behaviors and weight gain; these deserve more attention. Finding time and energy for exercise may be challenging after a physically or emotionally fatiguing work day; difficulty in balancing work with family demands, especially common for working women, may exacerbate this [61]. Beyond its behavioral effects, chronic stress also leads to deposition of intra-abdominal fat [62]. Comfort eating, as well as other unhealthy behaviors, serves as coping strategies for many workers to better tolerate or relieve work-induced stress [63–65]. Intention to exercise is also often disrupted [66, 67]. Shiftwork and excessively long work hours disrupt sleep and metabolism [68–71], in turn increasing the risk of obesity and metabolic syndrome [72]. Shiftwork interferes with exercise through physiological as well as behavioral mechanisms [73].

### 4.2. Strengths and Weaknesses of the Study

The present study is cross-sectional, so we cannot determine the temporal direction of these associations: that is, did exposure to stressful working conditions predate the occurrence of smoking, overweight, or inactivity or merely play a role in their continuation. Another methodological weakness is that some individuals may have underestimated their body weight or smoking or overstated their exercise levels. Reassuringly, Huerta et al. reported that self-reported smoking has moderately good reliability [74]. BMI computed from self-reported data is underestimated by about one unit, with slightly larger effects in persons 60 years of age and little variation by ethnicity [75–77]. Such an effect here would likely have resulted in negligible information bias in the overall associations.

This study also has several important strengths. The limited range of job titles surveyed helped to reduce the likelihood of unmeasured confounding by other features of socioeconomic status. The high proportion of respondents who were nursing aides provided sufficient statistical power to confirm that these associations were observed even in the lowest SES group. Moreover, the high response rate helped to guard against selection bias and produced a sample with demographic characteristics representative of the entire company workforce (i.e., 200,000 people). Generalizability of the results is also strengthened by the fact that this sample of U.S. nursing home employees had health behaviors comparable to the national female workforce [78].

## 5. Conclusion

The health of healthcare workers, along with turnover and other consequences, is of high international concern. The job features implicated in this study are also known to be widespread in the healthcare sector. Nonetheless, there have been remarkably few studies examining workplace stressors and health behaviors specifically among healthcare workers. Frequent exposure to threats and violence and poor social climate at work predicted smoking relapse in nursing aides [26]. Workplace violence, job strain, and role conflicts also increased risk of poor sleep among nursing aides [47].

These findings suggest that WHP programs might benefit from recognizing and addressing the contribution of the work environment, whether direct or indirect, to the health and health behaviors of individual employees. In the present study, work experiences such as heavy lifting, assault, night work, low social support, and low decision latitude were all linked negatively to personal health behaviors. These aspects of the work environment thus appear to impact employee health indirectly as well as directly. Because these adverse working conditions are especially common for low-wage workers, it is reasonable to hypothesize that preventive efforts might also reduce some socioeconomic disparities in health.

Many workplace stressors are remediable through training, improved job design, and organizational changes [5, 34, 79–82]. However, far too few WHP programs address these important potential determinants of the very behaviors they seek to change. The U.S. Total Worker Health program (http://www.cdc.gov/NIOSH/twh/), within which this study was carried out, is one initiative to improve program design by encouraging greater sensitivity to the effects of working conditions [83, 84].

## Figures and Tables

**Table 1:** Self-reported working conditions and personal factors, by job title: 1,506 U.S. nursing home employees.

	Nursing aides (*n* = 836)^∗∗^	Other jobs^∗^ (*n* = 661)^∗∗^
*Physical requirements at work *		
Heavy lifting (%)	63	47
Rapid and continuous physical activity (%)	85	64
Awkward working postures (%)	75	55
Physically demanding work (%)	60	38
*Work organization *		
Low decision latitude (%)	27	25
High psychological demands (%)	91	88
Job strain (high demand, low control) (%)	25	21
Low schedule control (%)	21	20
Regular night shift (%)	25	20
*Social support at work *		
Low coworker support (%)	36	28
Low supervisor support (%)	25	17
*Safety and work climate *		
One or more assaults at work in the past 3 months (%)	51	32
Poor safety climate (%)	64	53
Employer tolerates discrimination (%)	21	16
*Work-family balance and second jobs *		
Imbalance between work and family life (%)	46	43
Low employer support for family or other personal responsibilities (%)	51	36
Having another paid job (%)	21	20
*Health behaviors and obesity *		
Current smoker (%)	27	21
Physically inactive (%)	24	22
Obese (BMI > 30) (%)	36	32
*Demographics *		
Age (mean ± SD)	38.8 ± 12.8	44.0 ± 11.9
Gender: female (%)	91	87

^∗^Licensed practical nurses (LPNs), registered nurses (RNs), physical and occupational therapists, office, laundry, food service, and janitorial staff.

^∗∗^Numbers of respondents vary slightly among rows due to missing values.

**Table 2:** Self-reported working conditions and personal factors, by age group: 1,506 U.S. nursing home employees.

	Younger than 40 years (n = 690)^∗^	40 years and older (n = 737)^∗^
*Physical requirements at work *		
Heavy lifting (%)	61	51
Rapid and continuous physical activity (%)	82	70
Awkward working postures (%)	74	59
Physically demanding work (%)	59	43
*Work organization *		
Low decision latitude (%)	26	26
High psychological demands (%)	92	87
Job strain (high demand, low control) (%)	23	23
Low schedule control (%)	23	17
Working at night (%)	22	23
*Social support at work *		
Low coworker support (%)	34	31
Low supervisor support (%)	22	20
*Safety and work climate *		
One or more assaults at work in the past 3 months (%)	48	38
Poor safety environment (%)	63	54
Employer tolerates discrimination (%)	19	18
*Work-family balance and second jobs *		
Imbalance between work and family life (%)	49	41
Low employer support for family or other personal responsibilities (%)	45	43
Having another paid job (%)	22	19
*Health behaviors and obesity *		
Current smoker (%)	24	26
Physically inactive (%)	23	23
Obese (BMI > 30) (%)	30	38

^∗^Number of respondents varied slightly among rows due to missing values.

## Abbreviations

BMI: Body mass index
CI: Confidence intervals
JCQ: Job content questionnaire
LPN: Licensed practical nurses
NA: Nursing aides
PR: Prevalence ratios
RN: Registered nurses
SAS: Statistical analysis system
SD: Standard deviation
SES: Socioeconomic status
WHP: Workplace health promotion.
